# Functional Neurological Symptoms After Mild Traumatic Brain Injury: A Scoping Review and Framework for Differentiating Functional and Organic Post-Concussion Presentations

**DOI:** 10.3390/life16060926

**Published:** 2026-06-01

**Authors:** Ioannis Mavroudis, Foivos Petridis, Alin Ciobîcă, Manuela Padurariu, Sotirios Papagiannopoulos, Dimitrios Kazis

**Affiliations:** 1 Faculty of Medicine, University of Leeds, Leeds LS2 9JT, UK; 2 Third Department of Neurology, Aristotle University of Thessaloniki, 54124 Thessaloniki, Greecedimitrios.kazis@gmail.com (D.K.); 3 Department of Biology, Faculty of Biology, Alexandru Ioan Cuza University of Iasi, Carol I Avenue 20th A, 700505 Iasi, Romania; 4 Center of Biomedical Research, Romanian Academy, Iasi Branch, Teodor Codrescu 2, 700481 Iasi, Romania; 5 Academy of Romanian Scientists, 3 Ilfov, 050044 Bucharest, Romania; 6 Preclinical Department, Apollonia University, Păcurari Street 11, 700511 Iasi, Romania; 7 “Socola” Institute of Psychiatry, Șoseaua Bucium 36, 700282 Iasi, Romania

**Keywords:** mild traumatic brain injury, concussion, functional neurological disorder, persistent post-concussion symptoms, functional cognitive disorder, psychogenic non-epileptic seizures

## Abstract

Persistent post-concussion symptoms (PPCS) following mild traumatic brain injury (mTBI) are common and frequently disabling. However, symptom persistence is often poorly correlated with injury severity or structural brain abnormalities. Increasing clinical and research evidence suggests substantial overlap between PPCS and functional neurological disorder (FND), yet this interface remains poorly synthesised and conceptually unresolved. To systematically review and synthesise the evidence linking mTBI with functional neurological symptoms, and to refine existing conceptual models by proposing a clinically useful framework for differentiating functional and organic contributions to persistent post-concussion presentations. A scoping review with narrative synthesis were conducted. Database searches yielded 120 records; after duplicate removal and abstract screening, 57 studies underwent full-text review. Included studies comprised systematic reviews, narrative and conceptual reviews, mechanistic hypothesis papers, primary observational studies, case series, case reports, and early interventional and neuroimaging investigations examining functional neurological symptoms in the context of mTBI. The literature demonstrates substantial phenomenological overlap between PPCS and FND across cognitive, motor, sensory, visual, and seizure-related domains. Functional neurological symptoms can emerge after concussion and may closely resemble PPCS, often in association with psychiatric comorbidity, dissociation, trauma exposure, and maladaptive attentional or illness-belief processes. Objective neurological impairment and injury severity show weak and inconsistent associations with symptom persistence. The evidence base is dominated by clinic-derived observational studies, with no population-level incidence estimates identified. Functional neurological symptoms represent a significant and under-recognised contributor to persistent symptoms after mTBI. Existing evidence supports moving beyond binary organic–psychogenic models toward a functional–organic differentiation framework that acknowledges dynamic interactions between injury-related and functional mechanisms. Improved screening, diagnostic communication, and stratified management are likely to enhance outcomes for patients with persistent post-concussion symptoms.

## 1. Introduction

Mild traumatic brain injury (mTBI), commonly referred to as concussion, represents one of the most frequent neurological injuries worldwide, accounting for the majority of traumatic brain injuries across civilian, sporting, and occupational settings [1,2,3,4]. Although most individuals experience symptom resolution within weeks, a substantial minority develop persistent post-concussion symptoms (PPCS), including headache, dizziness, fatigue, cognitive complaints, emotional disturbance, and sensory or balance symptoms that may persist for months or years [5]. The pathophysiology underlying PPCS remains incompletely understood, and there is growing recognition that persistent symptoms are often poorly correlated with conventional markers of injury severity or structural brain damage. Persistent post-concussion symptoms (PPCS) refer to the persistence of physical, cognitive, and emotional symptoms beyond the expected recovery period following mild traumatic brain injury, typically operationalised as symptoms lasting longer than 4–12 weeks depending on diagnostic criteria [5].

Traditionally, persistent symptoms after concussion have been conceptualised as the downstream consequence of neurometabolic disturbance, microstructural injury, or prolonged physiological recovery. However, the non-specific nature of PPCSs, their high prevalence in the general population, and their frequent overlap with psychiatric and functional disorders have long complicated causal attribution [6,7,8]. Symptoms commonly labelled as post-concussive—such as cognitive “brain fog,” fatigue, dizziness, and emotional lability—are not unique to mTBI and occur in a wide range of neurological and non-neurological conditions, as well as following non-head-injury trauma.

Early clinical studies highlighted that poor recovery after mTBI is often associated with factors other than injury characteristics alone [9]. Mooney and Speed (2001) demonstrated that psychiatric comorbidity, including depression, anxiety, dissociation, and conversion disorder, was strongly associated with prolonged recovery in a specialty mTBI clinic population, whereas objective neuropsychological impairment showed limited association with outcome [10]. Subsequent observational studies have reinforced the importance of affective, cognitive, and contextual factors in PPCS, including anxiety, fear-avoidance, maladaptive illness beliefs, and dissociative symptoms [11,12,13,14].

In parallel, Functional Neurological Disorder (FND) has been increasingly recognised as a common and disabling condition encountered in neurological practice. FND is characterised by genuine neurological symptoms arising from altered brain functioning rather than structural pathology, and diagnosis is based on positive clinical signs demonstrating internal inconsistency or incongruence with recognised neurological disease [6,15,16,17]. Functional symptoms span a wide range of phenotypes, including weakness, gait disturbance, tremor, sensory symptoms, speech and language disturbance, visual symptoms, and seizures. Cognitive symptoms have also been increasingly recognised, often conceptualised as Functional Cognitive Disorder (FCD) [18,19].

A growing body of evidence suggests substantial overlap between persistent symptoms after concussion and functional neurological presentations [20,21]. Systematic reviews have demonstrated a characteristic pattern of prominent subjective cognitive complaints with inconsistent or mild objective deficits across FND, fibromyalgia, and chronic fatigue syndrome, with similar profiles reported in mTBI and whiplash populations [22]. Broader systematic reviews of somatic symptom and related disorders further indicate that functional and somatic symptom burden is frequently elevated following mTBI, although methodological limitations and heterogeneity across studies preclude reliable incidence estimates [23].

Clinical reports increasingly describe functional neurological symptoms emerging after concussion. Case series and case reports have documented psychogenic gait disorders, functional weakness, visual conversion symptoms, dissociative presentations, and functional seizures complicating concussion recovery in both adult and pediatric populations [24,25,26,27,28]. More recently, a dedicated case series by Polich et al. (2024) described a broad spectrum of functional neurological phenotypes with symptom onset after concussion, many of which closely resembled PPCS, highlighting the diagnostic challenges inherent in routine clinical practice [29].

Among functional phenotypes, psychogenic non-epileptic seizures (PNES) have been particularly well studied in relation to head injury. Cross-sectional studies indicate that a history of traumatic brain injury—most commonly mTBI—is common in PNES populations and is associated with greater psychiatric comorbidity, trauma exposure, disability, and poorer functional outcomes [30,31,32]. Mechanistic syntheses have proposed that head injury may act as a precipitating or vulnerability factor for functional seizures through interactions between network-level disruption, stress responsivity, dissociation, and maladaptive learning processes, while emphasising that such associations are heterogeneous and not universally causal [33].

Beyond discrete diagnoses, emerging evidence suggests that persistent symptoms after concussion often reflect complex interactions between physical injury, psychological vulnerability, attentional processes, and contextual influences such as healthcare messaging and expectations. Narrative and conceptual reviews have argued against strict dichotomies between “organic” and “functional” pathology, proposing instead that concussion and FND may lie along a continuum of brain dysfunction shaped by injury, psychological factors, and social context [7,34,35].

Within this evolving landscape, Mavroudis et al. proposed the Functional Overlay Model, conceptualising persistent post-injury symptoms as arising from dynamic interactions between early injury-related changes and superimposed functional mechanisms [36]. In this framework, concussion acts as a salient trigger that may alter bodily sensations, cognitive efficiency, and emotional regulation, while functional processes—such as heightened symptom monitoring, altered predictive processing, and impaired sense of agency—may increasingly contribute to symptom persistence over time. The model offers a pragmatic way to understand mixed or evolving presentations, while acknowledging that organic and functional mechanisms may coexist [37].

Nevertheless, despite growing recognition of these interactions, the literature remains fragmented. Studies vary widely in design, definitions, populations, and outcomes, and few attempt to integrate functional neurological frameworks into concussion research in a systematic manner. Population-based incidence data for FND after mTBI are lacking, and there is limited synthesis across phenotypes, age groups, and clinical contexts. As a result, clinicians face ongoing uncertainty regarding how best to conceptualise, identify, and manage persistent symptoms after concussion.

The aim of the present study is therefore to provide a comprehensive scoping review and narrative synthesis of the evidence linking mild traumatic brain injury with functional neurological symptoms. By integrating data from systematic reviews, observational studies, case series, mechanistic papers, and emerging interventional research, this review seeks to clarify the scope of existing evidence, characterise the range of functional presentations reported after concussion, and identify key gaps that must be addressed to improve diagnosis, stratification, and treatment of patients with persistent post-concussion symptoms.

## 2. Materials and Methods

### 2.1. Study Design

This study was conducted as a scoping review with narrative synthesis, aiming to systematically identify, classify, and synthesise existing evidence examining the relationship between mild traumatic brain injury (mTBI), persistent post-concussion symptoms (PPCS), and functional neurological disorder (FND). The review focused on conceptual, clinical, mechanistic, and interventional literature, reflecting the heterogeneous nature of research at the intersection of concussion and functional neurological symptoms. As a scoping review, the objective was to map and synthesise heterogeneous evidence across conceptual, clinical, and mechanistic domains rather than to provide an exhaustive or quantitative systematic synthesis. Accordingly, study inclusion was guided by relevance and contribution to the conceptual framework rather than predefined numerical thresholds.

### 2.2. Search Strategy

A comprehensive literature search was conducted across major biomedical and psychological databases, including MEDLINE/PubMed, Embase, PsycINFO, and Scopus, from database inception to the most recent search date. The search strategy was intentionally broad to capture diverse study types relevant to this emerging interdisciplinary field. Search terms were developed to capture literature relating to mTBI and concussion in combination with functional neurological phenomena. Core search terms included combinations of:

- *mild traumatic brain injury* OR *concussion* OR *post-concussion syndrome* AND
- *functional neurological disorder* OR *conversion disorder* OR *functional cognitive disorder* OR *psychogenic non-epileptic seizures* OR *somatic symptom disorder*

Reference lists of included articles and relevant reviews were manually screened to identify additional eligible studies.

### 2.3. Eligibility Criteria

Studies were eligible for inclusion if they met the following criteria:

1. Involved human participants or provided clinically relevant conceptual or mechanistic analysis
2. Examined functional neurological symptoms, FND, or related constructs in the context of mTBI or concussion
3. Were published in peer-reviewed journals
4. Were available in English

Eligible study designs included systematic reviews, narrative and conceptual reviews, mechanistic hypothesis papers, observational studies, case series, case reports, and interventional or neuroimaging studies. Conference abstracts, non-peer-reviewed reports, editorials without substantive analysis, and animal-only studies were excluded.

### 2.4. Study Selection

The database search yielded 120 records. After removal of duplicate records, titles and abstracts were screened for relevance. Following abstract screening, 57 studies were retained for full-text review. Full texts were assessed for eligibility based on the predefined inclusion criteria, resulting in the final set of studies included in the review. Following full-text assessment, 47 studies met inclusion criteria (33 original studies and 14 review articles) and were included in the final synthesis.

The study selection process is summarised using a PRISMA-style flow approach, with studies further classified by evidentiary role (systematic reviews, primary studies, mechanistic papers, and case-based evidence).

### 2.5. Data Extraction

From each included study, the following information was extracted:

- Author(s) and year of publication
- Study design and evidentiary category
- Population characteristics (where applicable)
- Type of functional neurological presentation (e.g., cognitive, motor, sensory, seizure-related)
- Key findings relevant to the relationship between mTBI, PPCS, and functional symptoms

Data extraction focused on qualitative synthesis rather than quantitative pooling, given the heterogeneity of study designs and outcomes.

### 2.6. Study Classification

Included studies were categorised into the following groups to facilitate synthesis:

1. Systematic reviews
2. Narrative or conceptual reviews
3. Mechanistic or theoretical papers
4. Primary observational studies
5. Case series and case reports
6. Interventional and neuroimaging studies

This classification informed the Results section and highlighted gaps in the current evidence base.

### 2.7. Data Synthesis

Given the heterogeneity of methodologies, outcomes, and study designs, a narrative synthesis approach was employed. Findings were synthesised thematically, focusing on phenomenological overlap between PPCS and FND, associated risk factors, mechanistic hypotheses, clinical implications, and emerging interventional evidence. No meta-analysis was performed due to the absence of sufficiently homogeneous quantitative data.

### 2.8. Risk of Bias Assessment

Formal risk-of-bias assessment was not performed, as the review incorporated diverse study designs including reviews, conceptual papers, and case-based evidence. Instead, methodological limitations and sources of bias within the existing literature are addressed narratively in the Discussion.

## 3. Results

### 3.1. Study Selection

**Figure 1:**
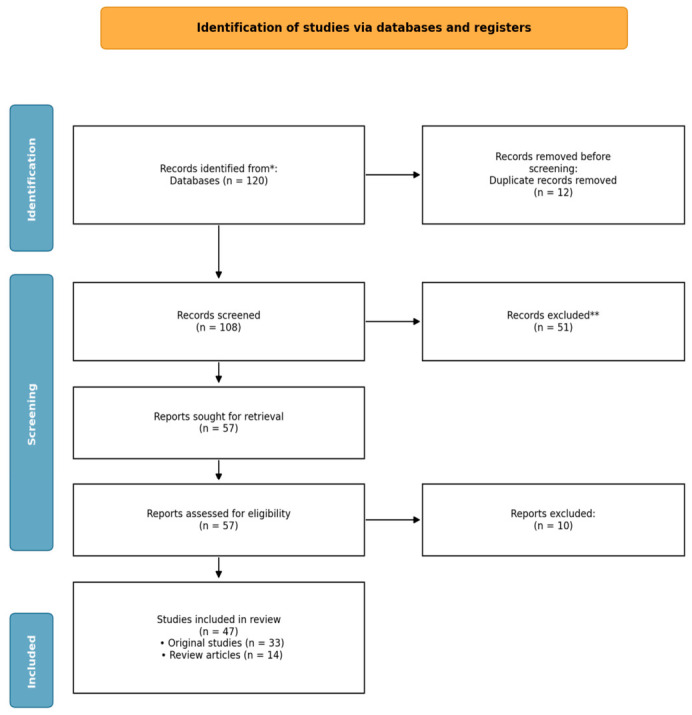
PRISMA flowchart of the research and study selection process.* databases searched include PubMed, Web of Science and Scopus, ** As per the exclusion criteria.

The electronic database searches yielded 120 records. After removal of duplicates, titles and abstracts were screened, resulting in 57 studies underwent full-text review, of which 47 met inclusion criteria and were included in the final synthesis (33 original studies and 14 review articles). Following full-text assessment, studies were excluded if they (i) did not address functional neurological symptoms in the context of mild traumatic brain injury (mTBI) or concussion, (ii) focused exclusively on moderate or severe TBI without relevance to concussion, or (iii) lacked primary data or substantive conceptual relevance (Figure 1).

After full-text screening, the final evidence base comprised systematic reviews, narrative and conceptual reviews, mechanistic hypothesis papers, primary observational studies, case series, case reports, and early interventional and neuroimaging studies, as summarised in Table 1. No population-based incidence studies of functional neurological disorder (FND) following mTBI were identified. Details of the study selection methodology are provided in the Methods section.

### 3.2. Overview of Study Types

The evidence base was heterogeneous and varied substantially in methodological strength. Systematic reviews and observational cohort studies provided the most robust data regarding associations between mTBI and functional neurological symptoms. In contrast, case reports and case series primarily served to illustrate clinical phenotypes and diagnostic challenges rather than establish causal relationships. Throughout the synthesis, higher weight was therefore given to systematic reviews and observational studies, while case-based evidence was interpreted cautiously as illustrative rather than confirmatory.

Two systematic reviews examined cognitive and somatic symptom profiles relevant to FND and mTBI [22,23]. Several narrative and conceptual reviews addressed diagnostic interfaces between concussion and FND [7,34,35]. One mechanistic hypothesis paper explored potential neurobiological vulnerability linking head trauma and functional seizures [33].

Primary data were provided by cross-sectional and clinic-based observational studies [10,11,12,30], supplemented by case series and case reports describing functional neurological presentations emerging after concussion [24,26,27,29]. Limited interventional and neuroimaging studies addressed treatment feasibility and neural correlates of functional cognitive disorder after concussion [38,39].

### 3.3. Systematic Reviews

#### 3.3.1. Cognitive Symptoms and Functional Cognitive Disorder

Teodoro et al. (2018) conducted a systematic review of cognitive outcomes across functional neurological disorder, fibromyalgia, and chronic fatigue syndrome, including 39 studies of FND [22]. Across conditions, the authors identified a consistent pattern of marked subjective cognitive complaints (e.g., forgetfulness, concentration difficulties, mental fatigue) with inconsistent or mild objective neuropsychological deficits. When objective impairments were reported, they most commonly involved attention, processing speed, and susceptibility to distraction.

Importantly, the authors explicitly noted that similar subjective–objective discordance has been reported in patients with mild traumatic brain injury and whiplash, suggesting overlapping cognitive mechanisms across these conditions. Performance validity testing indicated that poor effort accounted for symptoms in only a minority of patients, arguing against widespread feigning as an explanation for persistent cognitive complaints.

#### 3.3.2. Somatic and Functional Symptoms After mTBI

Jobin et al. (2025) [23] performed a systematic review of somatic symptom and related disorders (SSRD) in mTBI, screening over 6000 records and including 43 studies. Nine studies specifically examined functional seizures in relation to mTBI, while others evaluated somatization scales and clinician-diagnosed functional conditions [23].

Although the majority of included studies were rated as having unacceptable risk of bias, the acceptable-quality evidence consistently supported an association between mTBI and increased functional or somatic symptom burden. The authors highlighted the lack of high-quality prospective studies, and the absence of reliable incidence estimates for FND after mTBI.

### 3.4. Narrative and Conceptual Reviews

Phillips (2021) provided an expert narrative synthesis focused on FND in personal injury contexts [7]. The review emphasised that functional neurological symptoms commonly arise following minor accidents and injuries, including concussion, and cautioned against simplistic causal interpretations. Phillips highlighted the importance of diagnosing FND based on positive clinical signs, often demonstrated through inconsistency or distractibility, and stressed that such findings should not be equated with malingering.

More recent conceptual reviews proposed a continuum model linking concussion and FND, arguing that persistent post-concussion symptoms and functional neurological symptoms frequently overlap in phenomenology and underlying mechanisms [34,35]. These frameworks emphasised the role of expectations, illness beliefs, and healthcare interactions in shaping symptom persistence and recovery trajectories.

### 3.5. Mechanistic and Hypothesis-Driven Evidence

Popkirov et al. (2018) [33] explored the relationship between head trauma and psychogenic non-epileptic seizures (PNES). Reviewing existing epidemiological and neurobiological evidence, the authors noted that a history of head injury is frequently reported in PNES populations [33]. They proposed that even mTBI, despite normal routine imaging, may result in subtle disruptions of long-range network connectivity, potentially increasing vulnerability to dissociation and functional seizures in predisposed individuals.

The authors emphasised that such neurobiological vulnerability does not replace established psychological models of PNES but may interact with mechanisms such as stress responsivity, maladaptive learning, and illness attribution.

### 3.6. Primary Observational Studies

#### 3.6.1. Functional Seizures (PNES) and Prior mTBI

LaFrance et al. (2013) [30] examined patients with EEG-confirmed PNES in a cross-sectional cohort. Of the 92 patients included, 41 (44.6%) reported a history of traumatic brain injury, and 73.2% of these met criteria for mild TBI [30,31,32].

Compared with PNES patients without TBI, those with prior TBI demonstrated higher disability rates, lower global functioning, and significantly greater psychiatric comorbidity. After adjustment for age and sex, prior TBI was associated with increased odds of major depressive disorder, post-traumatic stress disorder, trauma or abuse history, and cluster B personality traits [30]. These findings suggest that PNES patients with a history of mTBI represent a subgroup with increased psychosocial vulnerability and functional impairment.

#### 3.6.2. Persistent Post-Concussion Symptoms and Psychiatric/Functional Factors

Mooney and Speed (2001) studied adults referred to a specialty mild TBI clinic, defining poor outcome as persistence of three or more post-concussive symptoms beyond three months [10]. Poor recovery was strongly associated with psychiatric comorbidity, including depression, anxiety disorders, and conversion disorder.

Dissociative symptoms were particularly prominent and robustly discriminated between good and poor recovery trajectories, with dissociation scores reported to predict outcome with high accuracy. In contrast, objective neuropsychological test abnormalities were not significantly associated with outcome, indicating that persistent symptoms were not primarily driven by measurable cognitive impairment [10].

Jobin et al. (2023) further demonstrated that, in patients with persistent post-concussion symptoms, greater functional neurological symptom severity was positively associated with higher post-concussion symptom burden, anxiety, and depression, reinforcing the interrelationship between functional symptoms and affective distress [12].

### 3.7. Case Series and Case Reports

Polich et al. (2024) reported a retrospective case series of 50 patients with clinician-confirmed FND whose functional neurological symptoms began after concussion [29]. Functional presentations were heterogeneous and included gait disturbances, functional seizures, speech and language symptoms, weakness, sensory symptoms, tremor, and visual or oculomotor disturbances.

Most patients exhibited multiple functional symptom types, and symptoms commonly overlapped with those typically labelled as persistent post-concussion symptoms, such as headache, dizziness, fatigue, and cognitive complaints [29].

Additional pediatric and adult case series and case reports described psychogenic gait disorders [24], conversion disorder with dissociative features [27], and functional motor deficits supported by advanced neurophysiological testing [26], illustrating the breadth of functional neurological presentations complicating concussion recovery across age groups.

### 3.8. Interventional and Neuroimaging Studies

Rioux et al. (2024) conducted a pilot randomised controlled trial evaluating the feasibility of cognitive-behavioural therapy tailored to functional cognitive disorder after concussion [38]. Both CBT and standard cognitive rehabilitation were well tolerated, with high adherence and credibility, although the study was not powered to detect differences in efficacy.

Westlin et al. (2025) examined structural brain measures in patients with functional cognitive disorder after concussion and reported no consistent group-level cortical or subcortical abnormalities compared with controls [39,40]. However, symptom severity and treatment response were associated with regional structural measures, suggesting that functional cognitive symptoms may relate to network-level vulnerability rather than focal injury.

**Table 1:** PRISMA-style classification of studies examining functional neurological symptoms after mild traumatic brain injury. This table summarises the studies included in the present review, classified by study design and evidentiary role. Included studies span systematic reviews, narrative and conceptual reviews, mechanistic hypothesis papers, primary observational studies, case series, case reports, and early interventional and neuroimaging investigations. The table highlights the heterogeneity of the existing literature and the predominance of clinic-based and observational designs. No population-based incidence studies of functional neurological disorder following mild traumatic brain injury were identified.

Category	Study	Year	Design	Population	Primary Contribution
**Systematic reviews**	Teodoro et al., *JNNP* [22]	2018	Systematic review	FND, fibromyalgia, CFS (39 FND studies)	Cognitive phenotype of FND/FCD; subjective–objective mismatch; mechanistic parallels with mTBI/whiplash
	Jobin et al., *Biopsychosoc Sci Med* [23]	2025	Systematic review	SSRD and mTBI (43 studies)	Evidence linking somatic/functional symptoms with mTBI; highlights poor methodological quality
**Narrative/conceptual reviews**	Phillips, *BMJ Neurol Open* [7]	2021	Expert narrative review	Injury-related FND (medicolegal focus)	Diagnostic reasoning, illness beliefs, positive-sign diagnosis after injury
	Burke & Silverberg, *Br J Sports Med* [34]	2025	Conceptual framework	Concussion ↔ FND continuum	Proposes unified conceptual continuum
	Mollica et al., *Semin Neurol* [35]	2025	Narrative review	PSaC & FND	Clinical intersections and rehabilitation implications
**Mechanistic/hypothesis papers**	Popkirov et al., *Seizure* [33]	2018	Mechanistic hypothesis	PNES after head trauma	“Dissociogenic lesion” concept; network vulnerability + psychological models
**Primary observational studies**	LaFrance et al., *Epilepsia* [30]	2013	Cross-sectional cohort	PNES with vs without TBI	Quantifies burden of TBI (mostly mTBI) in PNES; psychiatric and disability outcomes
	Mooney & Speed, *Brain Injury* [10]	2001	Clinic cohort	mTBI outpatients	Psychiatric comorbidity & dissociation predict poor recovery
	Jobin et al., *NeuroRehabilitation* [12]	2023	Cross-sectional	PPCS clinic sample	Association between FND severity, anxiety, depression, PPCS burden
	Picon et al., *J Psychosom Res* [11]	2021	Observational	PPCS patients	Psychological risk factors for functional/SSRD symptoms
**Case series**	Polich et al., *J Neuropsychiatry Clin Neurosci* [29]	2024	Retrospective case series	FND onset after concussion (n = 50)	Phenotypic spectrum and risk-factor clustering
	Otallah, *Pediatr Neurol* [24]	2020	Case series	Pediatric concussion	Psychogenic gait disorder complicating recovery
**Case reports**	Leczycki et al., *Cureus* [27]	2023	Case report	Adolescent mTBI	Conversion disorder with dissociation
	Jang & Seo, *Diagnostics* [26]	2019	Case report	mTBI	DTT/TMS used to support conversion diagnosis
**Interventional/feasibility studies**	Rioux et al., *BMJ Neurol Open* [38]	2024	Pilot RCT	FCD after concussion	CBT vs cognitive rehab feasibility
**Neuroimaging studies**	Westlin et al., *NeuroImage: Clinical* [39]	2025	Case–control imaging	FCD after concussion	Structural correlates of symptom severity and treatment response
**Meta-analysis**	Franekova et al., *Acta Neurol Belg* [19]	2025	Meta-analysis	Functional cognitive disorder	Metacognitive performance deficits across FCD; relevance to subjective–objective discordance in PPCS
**Narrative/conceptual reviews**	Stone et al., *Neurol Clin* [6]	2016	Narrative review with case examples	Functional disorders in neurology	Diagnostic principles for FND based on positive clinical signs; relevance to post-injury presentations
	Aaron & Buchwald, *Ann Intern Med* [20]	2001	Narrative review	Overlapping unexplained clinical conditions	Evidence for symptom overlap across functional somatic syndromes including post-traumatic presentations
	Pertab et al., *J Pers Med* [21]	2025	Narrative review/framework	Persisting symptoms after concussion	Autonomic, immune, and endocrine contributions to persisting PCS; integrative treatment framework
	Mavroudis et al., *Diagnostics* [1]	2022	Narrative review	PCS and chronic traumatic encephalopathy	Neuropathology, neuroimaging and fluid biomarker landscape in PCS
	Mavroudis et al., *Acta Neurol Belg* [5]	2025	Narrative review	Post-concussion syndrome	Epidemiology, pathophysiology, neuropathology, neuroimaging, and salivary biomarkers in PCS
	Mavroudis et al., *Acta Neurol Belg* [9]	2024	Narrative review	Personality traits in PCS	Personality and psychological risk profiles associated with persistent PCS
	Mavroudis et al., *Front Aging Neurosci* [18]	2025	Narrative review	Functional cognitive disorder	Diagnostic challenges, clinical features, and emerging directions in FCD
	Mavroudis et al., *Brain Sci* [36]	2025	Conceptual review	PCS and FND	Functional Overlay Model: diagnostic interfaces, risk mechanisms
	Mavroudis et al., *Brain Sci* [37]	2023	Conceptual review	Persistent PCS	Original Functional Overlay Model linking injury and functional mechanisms
**Primary observational studies**	McMahon et al., *J Neurotrauma* [2]	2014	Prospective cohort (TRACK-TBI)	Adult mTBI	Symptomatology and functional outcome trajectories after mTBI
	Theadom et al., *Br J Gen Pract* [3]	2016	Longitudinal population study	mTBI (New Zealand population)	Population-level burden of persistent problems 1 year after mTBI
	Nelson et al., *JAMA Neurol* [4]	2019	Multicenter prospective cohort	mTBI at US Level I trauma centers	Recovery trajectories and outcome predictors after mTBI
	Donnell et al., *Clin Neuropsychol* [8]	2012	Cross-sectional	Psychiatric, mTBI, and comorbid groups	Incidence of post-concussion symptoms in psychiatric diagnostic groups vs mTBI
	Ponsford et al., *Neuropsychology* [13]	2012	Prospective observational	mTBI	Psychological and contextual predictors of post-concussive symptoms at 3 months
	Doroszkiewicz et al., *J Neurotrauma* [14]	2021	Long-term follow-up	Persistent PCS	Anxiety, depression, and quality-of-life outcomes in persisting PCS
	Max et al., *J Neuropsychiatry Clin Neurosci* [28]	2013	Prospective observational	Pediatric mTBI	Incidence of psychiatric disorders 6–12 months after pediatric mTBI
	Westbrook et al., *Epilepsia* [31]	1998	Observational	Nonepileptic seizures after head injury	Early evidence linking head injury with PNES
	Salinsky et al., *Neurology* [32]	2011	Cross-sectional cohort	PNES in US veterans	TBI history and psychiatric burden in veteran PNES population
	McWhirter et al., *J Psychosom Res* [41]	2011	Prospective unblinded cohort	Suspected stroke/functional weakness	Diagnostic accuracy of Hoover’s sign for functional weakness
	Daum et al., *J Neurol Neurosurg Psychiatry* [42]	2015	Pilot validation study	Conversion disorder	Interobserver agreement and validity of bedside positive signs
	Lagrand et al., *Eur J Neurol* [43]	2025	Prospective study (TASMAN)	Functional movement disorders	Positive history-based signs aiding early FMD diagnosis
**Case reports**	Foutch, *J Optom* [25]	2015	Case report	Adult patient	Atypical presentation of visual conversion disorder
**Neuroimaging studies**	Aybek et al., *PLoS One* [15]	2015	Case–control fMRI	Conversion disorder	Emotion–motor interactions implicated in functional motor symptoms
	Maurer et al., *Neurology* [16]	2016	Resting-state fMRI	Functional movement disorder	Impaired sense of agency network in FMD
	Diez et al., *J Neurol Neurosurg Psychiatry* [17]	2019	Multimodal imaging	FND	Corticolimbic fast-tracking: enhanced multimodal integration in FND
	Allendorfer et al., *Neuroimage Clin* [40]	2019	Case–control fMRI + biomarker	PNES vs healthy controls	Stress-response neural signature differentiating PNES from controls
**Interventional/feasibility studies**	Potter et al., *J Neurol Neurosurg Psychiatry* [44]	2016	Randomised waitlist-controlled trial	Persistent post-concussional symptoms	Efficacy signal for CBT in persistent PCS after predominantly mild–moderate TBI
	Scheenen et al., *J Neurotrauma* [45]	2017	Randomised trial	Adult mTBI	CBT intervention compared with telephone counselling early after mTBI
	Silverberg et al., *J Head Trauma Rehabil* [46]	2013	Pilot RCT	At-risk mTBI	Cognitive–behavioural prevention of PCS in at-risk patients
	Tomfohr-Madsen et al., *J Head Trauma Rehabil* [47]	2020	Pilot RCT	Adolescent persistent PCS	CBT for insomnia in adolescents with persisting PCS

## 4. Discussion

### 4.1. Principal Findings

This scoping review synthesises a heterogeneous but increasingly coherent body of evidence indicating substantial overlap between persistent post-concussion symptoms (PPCS) and functional neurological disorder (FND). Across systematic reviews, observational cohorts, mechanistic syntheses, and case-based reports, three principal findings emerge. First, persistent symptoms after mild traumatic brain injury (mTBI) frequently exceed what can be accounted for by objective neurological impairment or measurable injury severity, with psychiatric comorbidity, dissociation, trauma exposure, and maladaptive illness beliefs repeatedly identified as stronger predictors of poor recovery than injury parameters [10,12,13]. Second, functional neurological phenotypes—including functional cognitive disorder, psychogenic non-epileptic seizures, functional motor and gait disturbance, and functional sensory or visual symptoms—are reported across the post-concussion symptom spectrum, and often closely resemble the symptom profile of PPCS [24,25,26,27,28,29]. Third, no population-based incidence data for FND following mTBI were identified, and the existing evidence base is dominated by clinic-derived samples that may overestimate the prevalence of severe or refractory cases [23].

### 4.2. Phenomenological Overlap Between PPCS and FND

**Figure 2:**
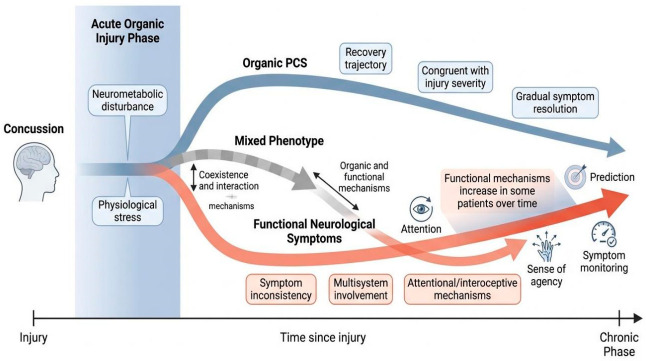
Conceptual schematic illustrating the proposed functional–organic differentiation framework for persistent post-concussion symptoms. Following an initial concussion, early organic and physiological processes may predominate in the acute and subacute phases. Over time, three broad clinical trajectories may emerge: (1) predominantly organic post-concussion symptoms with gradual recovery, (2) predominantly functional neurological symptoms characterised by multisystem involvement and altered attentional, predictive, and interoceptive processes, and (3) mixed presentations in which organic and functional mechanisms coexist and interact. The framework emphasises dynamic evolution over time rather than a binary distinction between organic and functional pathology.

The most striking finding across the included literature is the consistency with which subjective–objective discordance has been described in both PPCS and FND. Teodoro and colleagues (2018) reported that patients with FND, fibromyalgia, and chronic fatigue syndrome demonstrate prominent subjective cognitive complaints despite mild and inconsistent objective neuropsychological deficits, a pattern that the authors explicitly extended to mTBI and whiplash populations [22]. Similar patterns have been documented in PPCS cohorts, in which patient-reported symptom burden often outweighs objective findings on neuropsychological testing and neuroimaging [8,13]. Polich and colleagues (2024) further illustrated that FND symptoms beginning after concussion frequently coexist with—and are clinically indistinguishable from—symptoms typically labelled as post-concussive, including headache, dizziness, fatigue, and cognitive complaints [29]. Together, these data support the view that PPCS and FND should not be conceptualised as mutually exclusive diagnostic entities but as overlapping clinical presentations that may share common mechanistic substrates [34,35] (Figure 2).

### 4.3. Mechanistic Considerations

**Figure 3:**
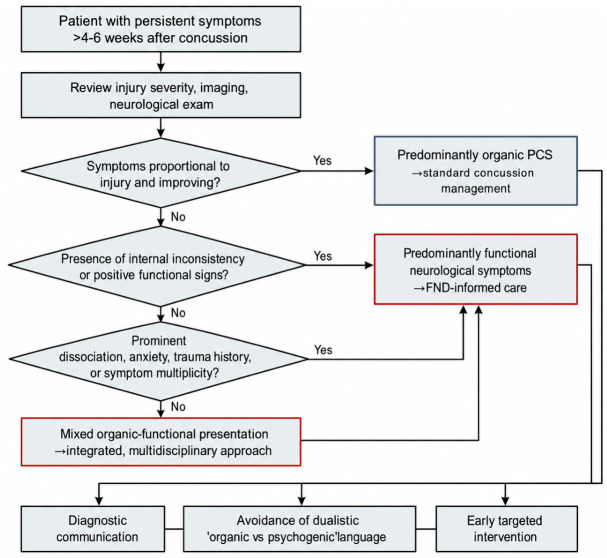
Flow diagram outlining a pragmatic clinical approach to patients presenting with persistent symptoms following concussion. The pathway integrates assessment of injury-related factors with targeted screening for functional neurological features, psychiatric comorbidity, and dissociative symptoms. Based on clinical findings, patients may be stratified into predominantly organic post-concussion symptoms, predominantly functional neurological symptoms, or mixed presentations, guiding appropriate management and referral pathways. The model underscores the importance of positive diagnostic features, careful diagnostic communication, and early targeted intervention.

The mechanistic literature reviewed offers complementary frameworks for understanding this overlap. Popkirov and colleagues (2018) proposed that mTBI may act as a “dissociogenic” event, producing subtle disruptions of large-scale network connectivity that interact with psychological vulnerability and stress responsivity to predispose susceptible individuals to functional seizures and other functional phenotypes [33]. Neuroimaging studies in FND populations have demonstrated altered emotion–motor coupling, impaired sense of agency, and enhanced corticolimbic integration, suggesting that functional symptoms arise from genuine alterations in brain function rather than from feigning or simulated deficits [15,16,17,40]. Westlin and colleagues (2025) reported no consistent group-level structural abnormalities in patients with functional cognitive disorder after concussion, although regional measures correlated with symptom severity and treatment response, supporting a network-vulnerability rather than focal-lesion model [39]. Collectively, these findings align with predictive-processing accounts of FND, in which heightened attentional focus on bodily symptoms, altered interoceptive predictions, and impaired sense of agency drive symptom generation and persistence (Figure 3).

### 4.4. Clinical Implications and the Functional–Organic Framework

The conceptual reviews included in this synthesis converge on the inadequacy of strict organic–psychogenic dichotomies. Burke and Silverberg (2025) and Mollica and colleagues (2025) argue that concussion and FND lie along a continuum of brain dysfunction shaped by injury, psychological factors, and contextual influences [34,35]. The Functional Overlay Model proposed by Mavroudis and colleagues operationalises this continuum by conceptualising persistent symptoms as arising from dynamic interactions between early injury-related changes and superimposed functional mechanisms, with their relative contribution evolving over time [36,37]. The framework presented in this review extends these concepts by proposing a functional–organic differentiation pathway: in the acute and subacute phases, organic processes predominate, but over time three broad trajectories may emerge—predominantly organic, predominantly functional, or mixed presentations. This framework has direct clinical implications. Positive diagnostic signs of FND, such as Hoover’s sign for functional weakness, distractibility in tremor, and inconsistency in cognitive testing, can be applied at the bedside in post-concussion patients [41,42,43], and early recognition opens the door to evidence-based treatments. Cognitive–behavioural interventions, both general and FND-specific, have demonstrated feasibility and preliminary signals of efficacy in PPCS and functional cognitive disorder after concussion [38,44,45,46,47], providing a tangible therapeutic target where conventional concussion rehabilitation may have plateaued.

### 4.5. Strengths and Limitations

This review has several strengths. Its broad scoping approach enabled integration of conceptual, mechanistic, observational, and interventional evidence across age groups and functional phenotypes, providing a more holistic synthesis than narrower reviews focused on single conditions. The classification of evidence by study design and evidentiary role permitted transparent weighting of conclusions, with greater interpretive weight assigned to systematic reviews and observational cohorts than to case-based illustrations. Several limitations must, however, be acknowledged. First, as a scoping review, no formal risk-of-bias assessment was undertaken, and the heterogeneity of designs and outcomes precluded meta-analysis. Second, the included literature is dominated by clinic-based samples drawn from specialty mTBI, FND, or epilepsy services, which are likely to overestimate symptom persistence and functional comorbidity relative to community samples. Third, no population-based incidence studies of FND after mTBI were identified, and reliable incidence estimates therefore remain unavailable. Fourth, definitions of FND, PPCS, and somatic symptom disorder varied substantially across studies, complicating direct comparison. Finally, the literature is restricted to English-language publications and is predominantly North American and European in origin, which may limit cultural generalisability.

### 4.6. Future Research Directions

Several priorities for future research can be identified. Population-based prospective studies of FND incidence after mTBI are urgently needed to establish reliable epidemiological estimates and to clarify the temporal relationship between concussion and the emergence of functional symptoms. Standardised diagnostic and outcome measures—incorporating positive FND signs, screening tools for dissociation and somatic symptoms, and validated measures of post-concussion symptom burden—would enable more meaningful comparison across studies. Mechanistic studies combining multimodal neuroimaging, autonomic and stress-response physiology, and detailed psychological phenotyping are likely to clarify the network-level and predictive-processing substrates of functional symptoms after concussion. Finally, adequately powered randomised trials of stratified interventions, including FND-specific cognitive-behavioural and physiotherapeutic approaches, are needed to translate conceptual advances into improved patient outcomes.

## 5. Conclusions

Functional neurological symptoms constitute a significant and under-recognised contributor to persistent symptoms after mild traumatic brain injury. The available evidence supports moving beyond binary organic–psychogenic models toward an integrated functional–organic differentiation framework that acknowledges the dynamic interaction of injury-related and functional mechanisms. Improved clinician awareness, systematic application of positive diagnostic signs, and stratified, mechanism-targeted treatment are likely to enhance outcomes for patients with persistent post-concussion symptoms.

## Data Availability

No new data were generated or analysed in this study. All data supporting the findings of this review are derived from previously published studies and are available within the cited articles.
